# Uncovering functional deterioration in the rhizosphere microbiome associated with post-green revolution wheat cultivars

**DOI:** 10.1186/s40793-025-00723-4

**Published:** 2025-06-08

**Authors:** Monique E. Smith, Vanessa N. Kavamura, David Hughes, Rodrigo Mendes, George Lund, Ian Clark, Tim H. Mauchline

**Affiliations:** 1 Sustainable Soils and Crops, Rothamsted Research, Harpenden, Hertfordshire, UK; 2 Department of Ecology, Swedish University of Agricultural Sciences, Uppsala, Sweden; 3 Intelligent Data Ecosystems, Rothamsted Research, Harpenden, Hertfordshire, UK; 4 Laboratory of Environmental Microbiology, Embrapa Environment, Jaguariúna, SP Brazil

**Keywords:** Wheat dwarfing, Wheat microbiomes, Heritage cultivars, Plant-microbe interactions, Green revolution breeding, Microbial functions

## Abstract

**Background:**

During the Green Revolution, one of the biggest developments of wheat domestication was the development of new cultivars that respond well to fertilisers and produce higher yields on shorter stems to prevent lodging. Consequently, this change has also impacted the wheat microbiome, often resulting in reduced selection of taxa and a loss of network complexity in the rhizospheres of modern cultivars. Given the importance of rhizosphere microbiomes for plant health and performance, it is imperative that we understand if and how these changes have affected their function. Here, we use shotgun metagenomics to classify the functional potential of prokaryote communities from the rhizospheres of pre-green revolution (heritage) cultivars to compare the impact of modern wheat breeding on rhizosphere microbiome functions.

**Results:**

We found distinct taxonomic and functional differences between heritage and modern wheat rhizosphere communities and identified that modern wheat microbiomes were less distinct from the communities in the surrounding soil. Of the 113 functional genes that were differentially abundant between heritage and modern cultivars, 95% were depleted in modern cultivars and 65% of differentially abundant reads best mapped to genes involved in staurosporine biosynthesis (antibiotic product), plant cell wall degradation (microbial mediation of plant root architecture, overwintering energy source for microbes) and sphingolipid metabolism (signal bioactive molecules).

**Conclusions:**

Overall, our findings indicate that green revolution breeding has developed wheat cultivars with a reduced rhizosphere effect. The consequences of this are likely detrimental to the development of microbiome-assisted agriculture which will require a strong rhizosphere selective environment for the establishment of a beneficial plant root microbiome. We believe our results are of striking importance and highlight that implementation of microbiome facilitated agriculture will benefit from deliberately incorporating the development of beneficial plant-microbiome interactions, alongside traditional yield traits, to advance sustainable wheat production.

**Supplementary Information:**

The online version contains supplementary material available at 10.1186/s40793-025-00723-4.

## Introduction

As a result of the Green Revolution, we benefit from a huge increase in cereal grain production. This was largely due to the widespread use of fertilisers and pesticides, the development of agricultural practices and the introduction of high-yielding cultivars. Modern cultivars are characterised by increased tillering, larger seed heads producing higher yields, but on shorter stems to prevent lodging, and they respond well to high fertilizer inputs. However, they rely on unsustainable levels of agrochemical inputs, including synthetic fertilisers, which are environmentally harmful [64].

While pre-green revolution cultivars, such as heritage or landraces cultivars, are mainly grown on marginal fields on organic farms [40, 44] they are still an important genetic resource for breeding programs [13, 23], especially since they show tolerance to extreme weather events and other stresses [41]. Wheat domestication gave little to no consideration to belowground processes,as such, we are only beginning to understand how wheat developments during the Green Revolution has impacted the interactions between roots and soil organisms six decades after the modern cultivars were introduced into agriculture [19, 53, 63].

The rhizosphere, i.e., the interface between roots and soil, harbours a dynamic community of microorganisms. These communities play an important role in how plants function, ranging from beneficial effects, e.g., aiding nutrient acquisition, growth promotion and plant defences [35, 38, 56], to harmful effects, e.g., pathogens such as *Gaeumannomyces tritici* causative agent of take-all disease in most cereals [45]. The assembly of rhizosphere communities are largely determined by the exudates and structure of plant roots [55, 65] which the microbiome itself can modulate [21, 46], therefore it is not surprising that the domestication of wheat has been shown to influence protist, bacterial, nematode and fungal rhizosphere communities [19, 53, 63]. The general trend in these populations is that wheat domestication has reduced selection processes in the rhizosphere. This is demonstrated by the root microbiome of heritage wheat harbouring more unique taxa and more complex microbiomes than the modern cultivars. Furthermore, modern cultivars can be enriched in fungal pathogens [20]. Similar trends have also been found in the domestication of other cereals such as barley [7], soybean [58], rice [61] and durum wheat [59]. While these findings have been important for our understanding of microbial community dynamics there is now a requirement to understand microbiome assembly and function for these resources to be harnessed in sustainable agriculture programmes with less dependence on fertilizer inputs [8, 17]. Phylogenetic marker gene profiles can hint at the function of these communities [19]. However, to obtain a comprehensive understanding of how green revolution wheat developments have impacted microbiome function, there is a requirement for holistic shotgun sequencing methods to be deployed.

Whole shotgun metagenomic sequencing provides a representation of the genomes present in each sample, allowing the inference of functional potential of a microbial community. This technique has been used to study the wheat rhizosphere microbiome and identify microbes that consume plant-derived carbon [14], to ascertain differences in microbial zinc-mobilisation genes between high and low zinc wheat cultivars [66], and the comparison of antibiotic resistance genes in the rhizospheres of common crops [71]. To date, shotgun metagenomics has not corroborated the wheat domestication-driven changes in rhizosphere communities found using phylogenetic marker gene studies. A previous attempt was made in Canadian wheat cultivars and found no effect of domestication, but this study sampled at wheat senescence [48], a time when the structure of the root microbiome of annual plants has been found to degrade and become dominated by saprophytes [33]. However, a similar study conducted in durum (tetraploid) wheat demonstrated that domestication led to a decline in gene diversity and a shift in microbial functional traits, particularly related to nutrient cycling [72]. As yet, the impact of the wheat developments during the green revolution on the vegetative stages of hexaploid bread wheat plants has not yet been explored using shotgun metagenomics.

Given the strong evidence from previous studies that wheat domestication impacts the rhizosphere community structure at a taxonomic level, we predict that the same impact will be observed for a range of functional genes in these communities. We tested this hypothesis by growing two heritage and two modern wheat cultivars alongside unplanted bulk soil control pots under glasshouse conditions. At flowering stage, we sampled the rhizosphere soil and bulk soil samples and generated, analysed, and compared the shotgun metagenomic profiles, in terms of taxonomy and function, of the prokaryote communities from these sample types.

## Methods

### Wheat cultivars and growth conditions

Wheat cultivars (Table 1) were selected based on previous results by Kavamura et al. [19], which indicated differences in the root microbiome structure and predicted function between modern and heritage cultivars. Soil was collected from Stackyard bare-fallow soil mine (52.002997°N, 0.613058°W) in January 2019. Soil details are previously described [50]. Soil was sieved (2 mm mesh), mixed thoroughly, and stored at 4 °C in polythene bags prior to use. Seeds were obtained from a field trial at Rothamsted Research, U.K., described in Lovegrove et al. [29]. Seeds were surface sterilized as described previously (Reid 2021,70% ethanol, 10 min; 1.5% active chlorine, 1 h; 5 × rinse, sterile distilled water; overnight imbibition in sterile water at 4 °C), to prevent compounding effects of microbial communities transferred from parents or during processing. Seeds were then transferred to pre-soaked (sterile distilled water) filter paper in Petri dishes and germinated for three days in the dark at room temperature. Seedlings were transplanted to individual wells on a seed tray (1 × seedling per well) in Stackyard bare fallow soil and grown in a glasshouse at Rothamsted Research for two weeks (20 °C, 16 h/day light regime, watered daily) before vernalization for twelve weeks (4 °C; 8 h light and 16 h dark). After this time, 3 seedlings were transplanted to 6-inch diameter pots (approximately 1 kg soil per pot), with NPK granules [15% N, 9% P_2_O_5_, 11% K_2_O, 2% MgO with micro-nutrients (B, Cu, Fe, Mn, Mo, and Zn); Osmocote, United Kingdom] (∼5 g per pot) added to the soil surface of each pot. Five replicate pots were prepared for each wheat variety, and three unplanted bulk soil control pots were also set up using the same soil and fertilisation. Plants were grown in a glasshouse (20 °C, 16 h/day light regime) and watered daily or as required with tap water.

**Table 1:** Characteristics of the cultivars used in this study including the year of release, pedigree, ancestry and the presence of dwarfing genes

Cultivar	Year	Pedigree	Ancestry	Dwarfing gene
Chidham white chaff	1790	Not recorded	Heritage	No
Red lammas	1850	Not recorded	Heritage	No
Malacca	1997	Riband*(Rendezvous)*Apostle	Modern	*Rht2*
Gallant	2009	(Malacca*Charger)*Xi-19	Modern	*Rht2*

*Table was modified from Kavamura et al. [19] and Lovegrove et al. [29]

Pots were harvested at the start of flowering (Zadoks growth stage 61; approximately 10 weeks growth post vernalisation; [73]), resulting in twenty rhizosphere samples and three bulk soil samples. Loose soil from the root system of a given pot was carefully removed. A 10 g subsample of root system was transferred to a 50 ml Falcon tube and 30 ml sterile water added. Next, samples were shaken vigorously for 10 min using an orbital shaker to release rhizosphere soil. After this time 4 ml soil suspension was centrifuged (2 min, RT, 15,000 rpm), supernatant discarded, and remaining soil was flash frozen in liquid nitrogen and stored at − 80 °C.

### DNA extraction and sequencing

Genomic DNA was extracted from the bulk soil and rhizosphere soil sample (~ 0.25 g) using the Dneasy PowerSoil Pro kit (Qiagen, Venlo, Netherlands) and stored at − 80 °C. Extractions were performed according to the manufacturer’s instructions and with the use of the MP Biomedicals FastPrep-24 machine twice for the bead-beating step at 30 s at 5.5 m s^−1^. DNA purity and concentrations were measured with a NanoDrop 1000 spectrophotometer (Thermo Fisher Scientific, Wilmington, DE, United States), as well as a Qubit 2.0 Fluorimeter using the dsDNA HS assay kit (Thermo Fisher Scientific). 0.6 µg of DNA for each sample was sent to Novogene (UK) Company Limited for library preparation and sequencing using Illumina NovaSeq 6000 platform (HWI-ST1276) using a 150 bp paired-end sequencing strategy. An average of 2.65^8^ raw paired reads, ranging between 1.66^8^ – 3.46^8^) per sample was obtained.

Raw reads were processed with fastx_artifacts_filter (http://hannonlab.cshl.edu/fastx_toolkit/index.html; v0.0.14) to remove sequencing artefacts, and Trimmomatic (http://www.usadellab.org/cms/?page=trimmomatic; v0.39; [4]) with a minimum length of 80 bp. Quality checked reads were assigned to taxa using DIAMOND (https://github.com/bbuchfink/diamond; v2.0.13; [6]) and AnnoTree (http://annotree.uwaterloo.ca/annotree/app/downloads.html; v1.2; [15, 39]) for prokaryote identification. KEGG Orthology (KO) molecular functional identifiers were assigned by MEGAN6 Ultra (https://software-ab.cs.uni-tuebingen.de/download/megan6/welcome.html; v6.24.23; [3, 18]) and used to extract individual gene sequences for each functional KEGG identifier. Thus, processing resulted in two abundance tables for further analysis, one where reads were aligned to prokaryote taxa and another to functional genes.

### Statistical analysis

#### Data processing

Taxa and functional genes were removed if their total count was < 10 reads. Only taxa assigned to Phyla or lower were kept for further analysis and reads unclassified to functional genes were removed. This resulted in 5,908,371,442 reads for the taxonomy table and 4,392,129,033 for the functional table and in both cases samples with the most reads roughly double those with the lowest number of reads.

All statistical analysis and visualisation of results was done in R version 4.3.3 [49]. Alpha and beta diversities were calculated from rarefied data [68] while differential abundance analysis was done using DESeq2 variance stabilisation technique [36] to normalise taxonomy and function abundance tables. The rarefied tables were calculated by normalizing sequence number to minimum sample size (159,120,960 and 118,292,681 for taxonomy and function tables respectively) by random subsampling without replacement using *rarefy_even_depth* function in the phyloseq package version 1.46 [37]. Rarefaction curve analysis was used to test that the subsampling of sequences still yielded sufficient resolution of prokaryote communities and their functional genes (Fig. S1).

#### Alpha and beta diversity

To test whether the sample type, including heritage wheat rhizosphere, modern wheat rhizosphere and bulk soil, impacted alpha diversity, we obtained observed and Chao1 richness and Shannon diversity from the rarefied taxonomy and functional tables using *estimate_richness* function in the phyloseq package. Normality and homogeneity of variances of alpha diversity measures were tested before performing type 3 one-way ANOVA to account for unbalanced design due to fewer replicates in the bulk soil samples, and Tukey’s honest significant differences (HSD) with sample type as a main factor.

Sample differences of the rarefied taxonomy and functional tables were visualised with principal coordinate analysis (PCoA) plots using Bray Curtis dissimilarity. To statistically test for differences between sample types we used Permutational Multivariate Analysis of Variance (PERMANOVA) with Bray Curtis distance matrices using *adonis2* in the vegan package version 2.6–4 [42] with 9999 permutations. Pairwise comparisons of each group were evaluated with *pairwise.adonis* using a false discovery rate correction for multiple tests. We evaluated differences among sample variability using homogeneity of multivariate dispersions tests (*betadisper*), followed by ANOVAs to compare the mean distance-to-centroid. Pairwise dispersion comparisons were carried out using Tukey’s HSD.

#### Differential abundance analysis

Differential abundances of individual 21,805 taxa and 10,189 functional genes were calculated using DESeq2 package version 1.42.1 [28] which is particularly powerful for small datasets [24]. Maximum-likelihood estimates for the log2-fold change between conditions associated with each gene or taxa were calculated using a negative binomial generalized linear model. Contrasts between each level of sample type were made and tests of significance were conducted using Wald’s test, employing α = 0.05 and a Benjamin–Hochberg false discovery rate (q) of 0.05 to control type I error rate in the face of multiple comparisons. Bayesian adaptive shrinkage was then applied to reduce the log2-fold change towards zero for taxa or genes with low mean counts or a high dispersion in their count distribution [60]. We then considered taxa or genes to be enriched in a particular sample type only if the resulting shrunken log2-fold changes were > 1 or < −1, i.e. double in abundance [54, 57].

Differential abundance analysis was done on all genes but for visualisation we only showed those that were considered enriched in a particular sample type and we compared the results of the analysis from these against ten housekeeping genes used as a baseline. These housekeeping genes were selected to cover different parts of the genome and that encode proteins involved in different metabolic activity, except for the ribosomal genes, similar to the gene selection in multilocus sequence typing (MLST; [30, 62]). These included signal recognition particle protein (*ffh*), glutamine synthetase (*glnA*), DNA gyrase (*gyrB*), transcription termination factor Rho (*rho*), 50S ribosomal protein L9 (*rplI*), 50S ribosomal subunit protein L17 (*rplQ*), RNA polymerase (*rpoZ),* DNA topoisomerase I (*topA*), nucleoside-specific channel-forming protein (*tsx*), ATP synthase F1, β-subunit (*atpD*).

## Results

### Alpha diversity

**Fig. 1:**
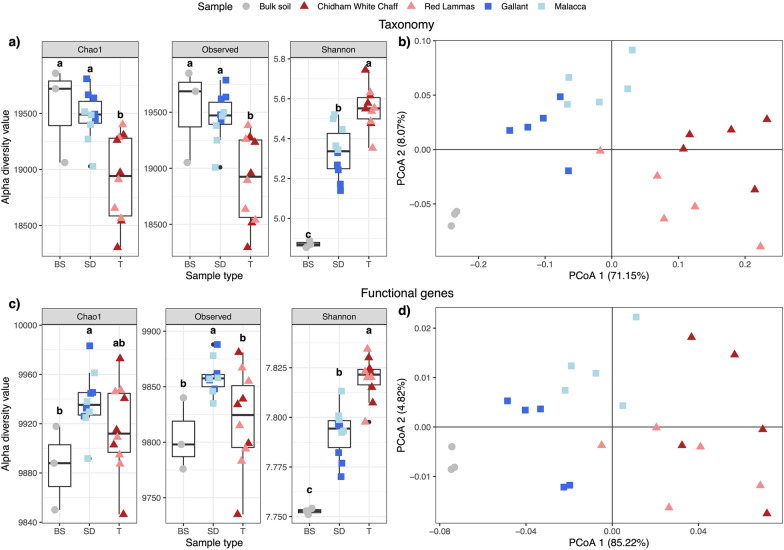
Diversity measures of the taxonomy and function of prokaryote communities. Alpha diversity is represented in plots (**a**) and (**c**), and Principal Coordinate Analysis (PCoA) plots based on Bray–Curtis dissimilarity demonstrate beta diversity in plots (**b**) and (**d**), for taxonomy and function respectively. Significant differences are shown between sample types, bulk soil samples (circles) and the rhizospheres of modern (squares) and heritage (triangles) wheat whereby sample types with the same letter are not statistically different from each other. Colours differentiate between the wheat different cultivars

Overall, alpha diversity differences were statistically significant between sample types, i.e., bulk soil and rhizosphere soil from modern and heritage cultivars, for both taxonomy and function of the prokaryote communities (Fig. 1a, c). Heritage cultivars harboured fewer taxa (observed and chao1) in their rhizospheres than bulk soils and modern rhizospheres; however, when rarefied read abundances were considered, their communities were the most diverse (Shannon’s species diversity). Functional genes followed a similar pattern, though the differences were less profound. Shannon’s diversity was marginally greater in heritage rhizospheres and more genes ascribed to modern than heritage rhizospheres. Wheat rhizospheres regardless of cultivar were found to have a higher functional diversity than bulk soil samples.

### Beta diversity

We found very similar PERMANOVA results for both taxonomy and function-assigned genes, whereby a high proportion of variation between samples was explained by sample type (bulk soil, modern and heritage wheat rhizospheres; Table 2). Pairwise comparisons between sample types were all significantly different with the highest R^2^ between heritage rhizosphere and bulk soils. This difference is partly explained by significantly different dispersions between groups for taxonomy and function (ANOVA, F = 11.9, *p* < 0.001; F = 6.8, *p* = 0.006, respectively). *Posthoc* comparisons revealed that all groups differed in dispersion for taxonomy, but for function, there was less dispersion in the bulk soil samples compared to modern (*padj* = 0.056) and heritage (*padj* = 0.004) rhizospheres. Nevertheless, the PCoA clearly supports the PERMANOVA results that sample type is an important factor in determining the prokaryote taxonomy and function since they were clustered separately in multivariate space (Fig. 1b, d). Most of the variation was explained by PCoA 1 for both taxonomy and functional datasets which demonstrated that, from the cultivars included here, modern wheat cultivars harbour prokaryote community profiles that are more similar to bulk soil than heritage cultivars do. The shift in the prokaryote community was represented by an increasing relative abundance of Pseudomonadota (Proteobacteria) and Bacteroidota from bulk soil to rhizosphere soil of modern and heritage wheats and a higher proportion of Acidobacteroidota in bulk soil samples (Fig. S2).

**Table 2:** PERMANOVA and Tukey’s pairwise results for both taxonomy and function of Prokaryote communities. All combinations of sample type, bulk soil (BS) and rhizosphere soil of modern (M) and heritage (H) cultivars, were present. Significant differences in bold, i.e., *p < 0.05,* and *p* values of pairwise tests have been adjusted for multiple comparisons using a false-discovery rate correction

Variable/	Taxonomy			Function		
Pairwise comparison	R^2^	F	*p*	R^2^	F	*p*
Sample type	0.63	21	** < 0.001**	0.72	25.6	** < 0.001**
BS vs M	0.55	13.7	**0.004**	0.59	15.5	**0.004**
BS vs H	0.66	21.5	**0.004**	0.76	34.9	**0.004**
M vs H	0.46	15.6	** < 0.001**	0.56	23.3	** < 0.001**

Bold indicates Welch Two Sample tests (*p* < 0.05) using a false-discovery rate correction to adjust for multiple comparisons

### Differential abundance

**Fig. 2:**
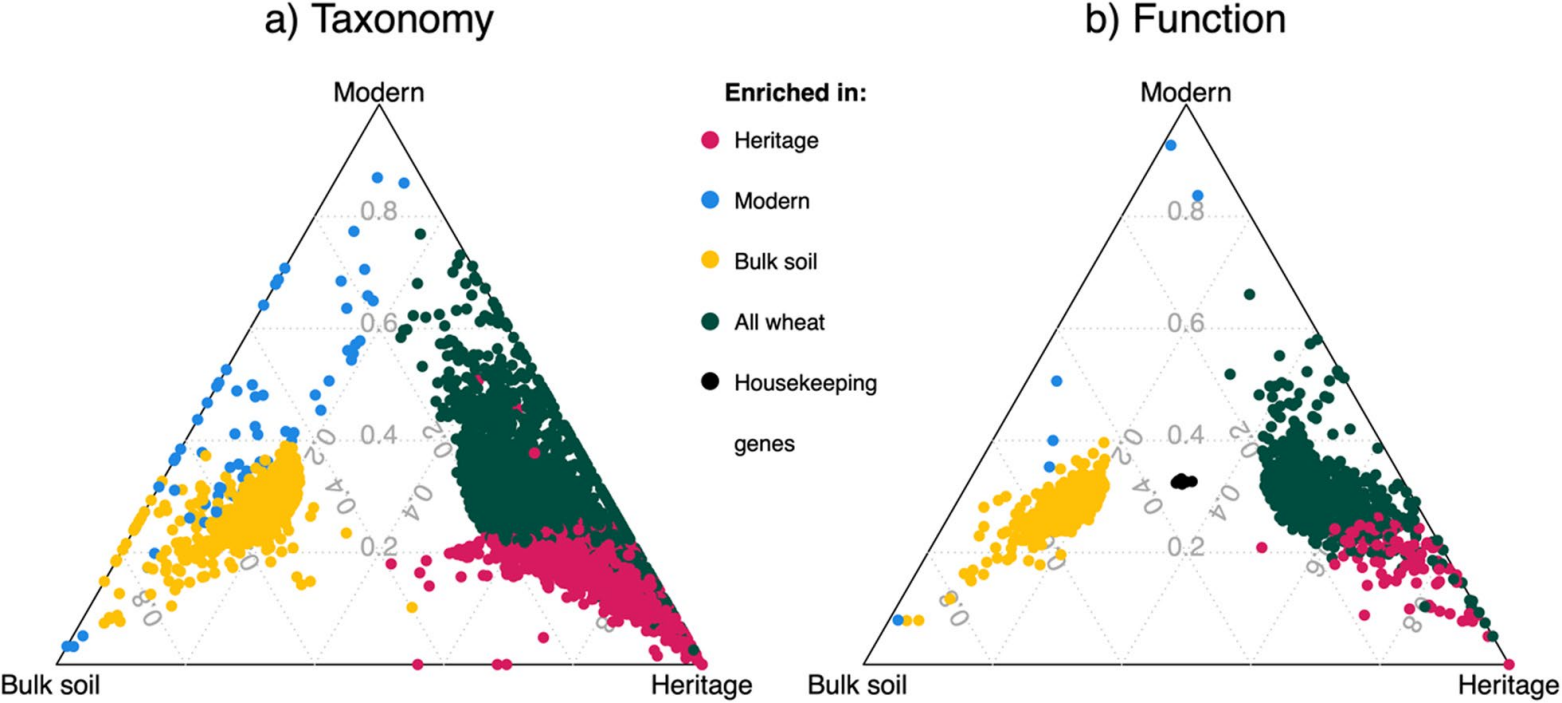
Ternary plots of differentially abundant taxonomy (**a**) and function (**b**) of prokaryote communities. Each point represents a taxa (**a**) or functional gene (**b**) and their position represents the contribution of the indicated sample type, bulk soil, modern and heritage rhizospheres, to the total normalised abundance. Taxa or functional genes are significantly different in abundance for different contrasts (Ward *p* < 0.05, FDR < 0.05, shrunken log fold changes > 1) and their colours indicate the group they are enriched in (i.e., higher abundance). Taxa or functional genes enriched in ‘all wheat’ samples, i.e., rhizosphere samples regardless of ancestry, were determined by contrasts with bulk soil samples and vice versa for enriched in bulk soil. Enriched in heritage rhizospheres was determined compared with modern rhizospheres, regardless of bulk soil and vice versa for modern. Therefore, some points could be characterised by two colours but the comparison between heritage and modern rhizospheres is the one presented as this is the most relevant for our hypothesis. Prokaryote housekeeping genes were included in b as a reference for differentially abundant functional genes (see methods for a list of housekeeping genes)

Out of 21,805 taxa and 10,189 functional genes, 5072 (23%) and 1719 (17%) respectively were differentially abundant for at least one contrast between the sample types (Fig. 2; Ward *p* < 0.05, FDR < 0.05, shrunken log2 fold > 1 or <  − 1).These differentially abundant taxa and functional genes make up 46% and 5% of total raw reads, respectively. The primary aim of this work is to compare abundances of genes between wheat ancestral genotypes. However, the biggest contrast was between heritage rhizosphere and bulk soil prokaryote communities whereby 4975 taxa and 1712 functional genes were differentially abundant. Modern rhizosphere communities were more similar to bulk soil communities than the heritage rhizosphere communities with 1114 taxa and 194 functional genes differentially abundant, 78 and 89% less than the heritage vs bulk soil contrast, respectively. Despite this difference, the taxa that were enriched in either the heritage or modern wheat rhizosphere samples, compared with the bulk soil, belonged mostly to the same three phyla, Pseudomonadota, Bacteroidota and Actinomycetota (Actinobacteria), which when combined, accounted for 95% and 92% of the enriched taxa respectively (Fig. S3). Bulk soil samples were enriched in taxa from a wide range of phyla when compared to both rhizosphere types, dominated by Bacillota (Firmicutes), but the highest proportion of taxa belonging to various rare phyla (phyla that contain less than 5% of all enriched taxa). Interestingly, 19 of the 20 most abundant functional genes that were enriched in heritage rhizospheres relative to bulk soil samples, were also more abundant in heritage than modern rhizosphere samples (ANOVA, *p* < 0.05; Table S1). The one exception being K07305 (peptide-methionine (R)-S-oxide reductase) which was equally abundant in the rhizosphere of heritage and modern but has low representation in bulk soil samples. It was found that 13 genes out of these 20 most abundant genes were also more abundant in modern rhizospheres compared to bulk soil samples.

**Fig. 3:**
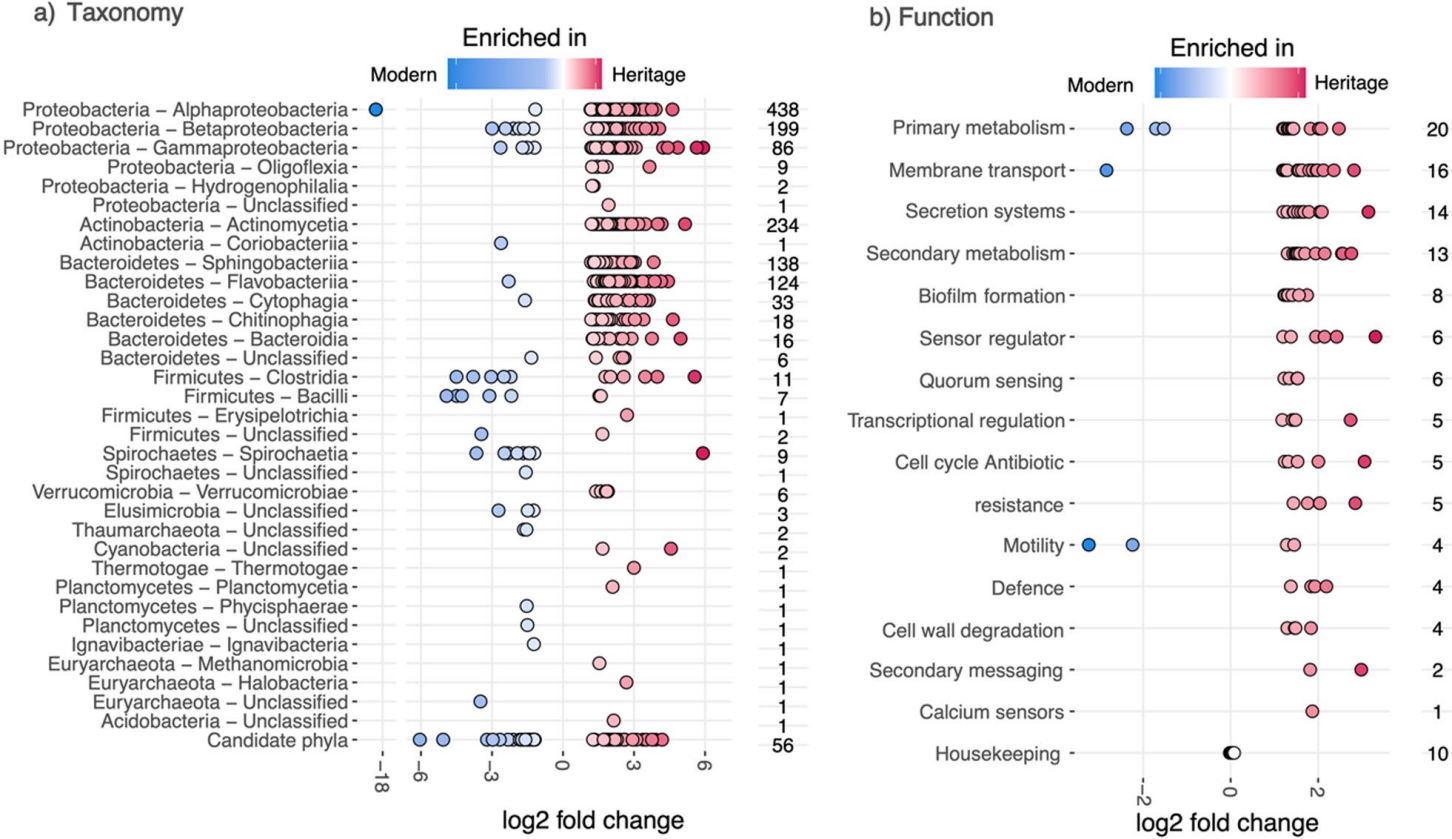
Differentially abundant taxonomy (**a**) and function (**b**) of prokaryote communities between modern and heritage wheat rhizospheres. Taxa and functional genes have been assigned to Class or functional group (based on KEGG orthology) respectively and the number of differentially abundant taxa or functional genes in each group are listed to the right of each plot. Only significant (Ward *p* < 0.05, FDR < 0.05,) and large (at least double; shrunken log2 fold > 1 or < −1 differences are shown except for the 10 housekeeping genes included for reference against the differentially abundant functional genes

There were 1414 taxa and 113 functional genes that were differentially abundant between the modern and heritage rhizosphere communities, most of which (95%) were enriched in the heritage rhizosphere communities (Fig. 3). The taxa enriched in the heritage rhizosphere communities followed the same pattern as when compared to bulk soil whereby 96% of reads were from Pseudomonadota, Bacteroidota and Actinomycetota (Fig. S3). The modern rhizosphere communities were mostly enriched in Bacillota, Pseudomonadota, Spirochaetota and the highest proportion of enriched taxa belonged to rare phyla (phyla that contain less than 5% of all enriched taxa). The differentially abundant functional genes belong to 15 different functional groups whereby heritage rhizospheres had enriched genes belonging to each group (6,574,723 raw reads) and the six functional genes enriched in the modern rhizospheres belonged to three categories (3257 raw reads; Fig. 3b). Of the 107 genes enriched in the rhizospheres of heritage wheats, 40% of reads mapped to genes associated with secondary metabolism, 21% with plant cell wall degradation, 10% with two-component sensor regulation, 8% with membrane transport, 12% with primary metabolism (other than cell wall degradation), 2% with secretion systems, 2% to transcriptional regulation and the remaining 5% to quorum sensing, cell cycle, biofilm formation, antibiotic resistance, motility, defence, and secondary messaging functions (Table 3).

**Table 3:** Summaries of the taxa assigned to the 113 functional genes identified as differentially abundant between heritage and modern wheat rhizospheres. The functional genes are identified by the Kyoto Encyclopaedia of Genes and Genomes identifier (KEGG ID) and put into broad functional groups. The total read count refers to the total number of reads for each functional gene found in bulk soil and rhizosphere samples normalised by DESeq2 size factors for each sample. The proportion of reads belonging to either heritage or modern rhizosphere samples are indicated and genes enriched in the heritage and modern rhizospheres are separated by the horizontal line. Reads were reassigned to the taxa containing that gene and richness was calculated as the observed richness, i.e., the total number of taxa in either tall or semi-dwarf samples containing the corresponding functional gene, and Shannon’s diversity is the richness weighted by normalised read count to consider the evenness of the community. Numbers in bold represent significant differences between the means of either richness or diversity between heritage and modern rhizosphere samples (n = 20) as determined by Welch Two Sample t-tests (*p* < 0.05) using a false-discovery rate correction to adjust for multiple comparisons

KEGG ID	Function	Gene name	Total read count	Proportion of read count		Observed richness (mean ± sd)		Shannons diversity (mean ± sd)	
			(Normalised)	Modern (%)	Heritage (%)	Modern	Heritage	Modern	Heritage
K14266	Secondary metabolism	tryptophan 7-halogenase [EC:1.14.19.9]	2,417,360	27	71	**174 ± 19**	**243 ± 19**	**3.5 ± 0.1**	**3.7 ± 0.1**
K16397	Secondary metabolism	epothilone polyketide synthase D	29,204	23	72	11 ± 2	11 ± 2	**1.8 ± 0.1**	**1.5 ± 0.3**
K16403	Secondary metabolism	O-methyltransferase	4801	22	74	5 ± 1	6 ± 1	**1.3 ± 0.2**	**1.0 ± 0.1**
K14627	Secondary metabolism	dehydratase [EC:4.2.1.-]	3362	22	75	**5 ± 2**	**10 ± 2**	**1.3 ± 0.3**	**1.7 ± 0.2**
K14368	Secondary metabolism	3-alpha-mycarosylerythronolide B desosaminyl transferase [EC:2.4.1.278]	2491	21	74	**4 ± 1**	**7 ± 1**	1.2 ± 0.3	1.4 ± 0.3
K11009	Secondary metabolism	murine toxin	2377	21	76	3 ± 1	4 ± 1	**0.9 ± 0.2**	**0.6 ± 0.4**
K19885	Secondary metabolism	dichlorochromopyrrolate synthase/catalase [EC:1.21.98.2 1.11.1.6]	1871	16	80	**3 ± 1**	**5 ± 1**	0.6 ± 0.4	0.9 ± 0.2
K21212	Secondary metabolism	NDP-hexose 2,3-dehydratase	1851	20	76	**3 ± 1**	**5 ± 1**	**0.8 ± 0.3**	**1.2 ± 0.3**
K16448	Secondary metabolism	methylation protein MtfA	1573	16	81	**2 ± 1**	**5 ± 1**	**0.6 ± 0.4**	**1.1 ± 0.2**
K15968	Secondary metabolism	tetracenomycin F2 cyclase [EC:4.2.1.154]	1019	19	76	**2 ± 1**	**4 ± 1**	**0.4 ± 0.4**	**1.1 ± 0.2**
K20086	Secondary metabolism	tryptophan oxidase VioA [EC:1.4.3.-]	203	8	91	**0 ± 0**	**2 ± 1**	**0.0 ± 0.0**	**0.2 ± 0.2**
K20090	Secondary metabolism	violacein synthase [EC:1.14.13.224]	191	8	92	**0 ± 0**	**1 ± 1**	0.0 ± 0.0	0.1 ± 0.3
K20088	Secondary metabolism	violacein biosynthesis protein VioE	125	10	84	**0 ± 0**	**1 ± 1**	**0.0 ± 0.0**	**0.3 ± 0.3**
K18578	Cell wall degradation	xyloglucan-specific exo-beta-1,4-glucanase [EC:3.2.1.155]	609,196	25	73	**120 ± 12**	**146 ± 10**	**3.7 ± 0.1**	**3.6 ± 0.1**
K18651	Cell wall degradation	oligoxyloglucan reducing-end-specific cellobiohydrolase [EC:3.2.1.150]	537,163	25	73	**112 ± 11**	**131 ± 9**	3.5 ± 0.0	3.5 ± 0.1
K18786	Cell wall degradation	cellobionic acid phosphorylase [EC:2.4.1.321]	75,143	20	78	**18 ± 4**	**24 ± 3**	**2.3 ± 0.1**	**2.0 ± 0.1**
K18576	Cell wall degradation	xyloglucan-specific endo-beta-1,4-glucanase [EC:3.2.1.151]	69,699	26	70	**28 ± 4**	**40 ± 5**	**2.5 ± 0.1**	**2.7 ± 0.1**
K01202	Primary metabolism	galactosylceramidase [EC:3.2.1.46]	322,927	27	68	**66 ± 10**	**83 ± 5**	3.1 ± 0.1	3.1 ± 0.1
K20455	Primary metabolism	2-methylcitrate dehydratase (2-methyl-trans-aconitate forming) [EC:4.2.1.117]	150,825	27	69	**24 ± 4**	**32 ± 3**	**2.1 ± 0.1**	**2.2 ± 0.1**
K02847	Primary metabolism	O-antigen ligase [EC:2.4.1.-]	101,796	27	70	**54 ± 7**	**79 ± 12**	3.2 ± 0.1	3.2 ± 0.2
K08961	Primary metabolism	chondroitin-sulfate-ABC endolyase/exolyase [EC:4.2.2.20 4.2.2.21]	45,190	20	77	18 ± 3	20 ± 2	1.8 ± 0.1	1.9 ± 0.1
K08325	Primary metabolism	NADP-dependent alcohol dehydrogenase [EC:1.1.-.-]	36,347	26	71	**21 ± 4**	**30 ± 3**	**2.5 ± 0.2**	**2.6 ± 0.1**
K00211	Primary metabolism	prephenate dehydrogenase (NADP +) [EC:1.3.1.13]	33,022	25	73	**7 ± 2**	**9 ± 2**	0.6 ± 0.2	0.7 ± 0.2
K03181	Primary metabolism	chorismate lyase [EC:4.1.3.40]	26,153	27	69	**22 ± 3**	**26 ± 5**	**2.6 ± 0.1**	**2.4 ± 0.2**
K01085	Primary metabolism	glucose-1-phosphatase [EC:3.1.3.10]	13,928	24	73	**13 ± 3**	**17 ± 2**	**2.1 ± 0.2**	**1.7 ± 0.4**
K01355	Primary metabolism	omptin [EC:3.4.23.49]	10,723	25	75	**7 ± 2**	**13 ± 2**	**1.0 ± 0.4**	**1.8 ± 0.3**
K01819	Primary metabolism	galactose-6-phosphate isomerase [EC:5.3.1.26]	8410	24	72	**10 ± 1**	**13 ± 2**	2.1 ± 0.1	2.0 ± 0.1
K16215	Primary metabolism	2-ketoarginine methyltransferase [EC:2.1.1.243]	4920	22	73	6 ± 1	7 ± 2	1.3 ± 0.3	1.0 ± 0.2
K00998	Primary metabolism	CDP-diacylglycerol–-serine O-phosphatidyltransferase [EC:2.7.8.8]	4027	24	72	**4 ± 1**	**5 ± 1**	1.1 ± 0.2	1.1 ± 0.2
K21280	Primary metabolism	3-hydroxy-4-methylanthranilyl-[aryl-carrier protein] 5-monooxygenase [EC:1.14.13.223]	3575	15	84	2 ± 1	2 ± 1	**0.4 ± 0.3**	**0.0 ± 0.0**
K12455	Primary metabolism	CDP-abequose synthase [EC:1.1.1.341]	3208	17	80	5 ± 1	6 ± 1	**1.4 ± 0.2**	**0.7 ± 0.2**
K21239	Primary metabolism	virion DNA-directed RNA polymerase [EC:2.7.7.6]	3016	18	81	2 ± 1	2 ± 0	0.2 ± 0.2	0.1 ± 0.1
K19033	Primary metabolism	30S ribosomal protein S31	2812	27	68	**6 ± 2**	**9 ± 1**	**1.4 ± 0.2**	**1.9 ± 0.1**
K03397	Primary metabolism	indoleacetate–-lysine synthetase [EC:6.3.2.20]	413	10	88	**0 ± 0**	**1 ± 1**	0.0 ± 0.0	0.1 ± 0.2
K05874	Sensor regulator	methyl-accepting chemotaxis protein I, serine sensor receptor	483,244	25	72	**108 ± 18**	**178 ± 22**	**3.4 ± 0.1**	**3.8 ± 0.1**
K17060	Sensor regulator	two-component system, sensor histidine kinase AauS [EC:2.7.13.3]	76,045	28	70	**32 ± 5**	**45 ± 6**	3.0 ± 0.1	3.0 ± 0.2
K05876	Sensor regulator	methyl-accepting chemotaxis protein III, ribose and galactose sensor receptor	38,854	19	79	15 ± 2	24 ± 2	**2.2 ± 0.1**	**2.5 ± 0.2**
K19611	Sensor regulator	ferric enterobactin receptor	3275	9	91	**2 ± 2**	**4 ± 2**	0.4 ± 0.4	0.6 ± 0.5
K07786	Sensor regulator	MFS transporter, DHA2 family, multidrug resistance protein	2193	15	82	**3 ± 1**	**6 ± 1**	1.0 ± 0.3	1.1 ± 0.4
K06080	Sensor regulator	RcsF protein	572	5	95	**0 ± 0**	**1 ± 0**	0.0 ± 0.0	0.0 ± 0.1
K10235	Membrane transport	alpha-glucoside transport system ATP-binding protein	117,671	27	71	**40 ± 6**	**55 ± 6**	**3.0 ± 0.1**	**3.2 ± 0.1**
K16088	Membrane transport	outer-membrane receptor for ferric coprogen and ferric-rhodotorulic acid	107,196	22	77	**36 ± 12**	**64 ± 11**	2.7 ± 0.4	3.0 ± 0.2
K16210	Membrane transport	oligogalacturonide transporter	89,287	28	70	**30 ± 3**	**35 ± 5**	2.5 ± 0.2	2.5 ± 0.2
K02532	Membrane transport	MFS transporter, OHS family, lactose permease	58,021	19	79	**17 ± 1**	**24 ± 3**	2.3 ± 0.1	2.3 ± 0.2
K16552	Membrane transport	polysaccharide biosynthesis/export protein ExoF	34,802	26	72	**13 ± 4**	**20 ± 4**	**1.8 ± 0.3**	**2.2 ± 0.2**
K11734	Membrane transport	aromatic amino acid transport protein AroP	24,606	26	64	**22 ± 3**	**27 ± 2**	**2.6 ± 0.1**	**2.4 ± 0.2**
K08156	Membrane transport	MFS transporter, DHA1 family, arabinose polymer utilization protein	19,358	19	79	**12 ± 3**	**19 ± 3**	1.9 ± 0.3	1.7 ± 0.4
K10094	Membrane transport	nickel transport protein	12,104	21	78	**10 ± 2**	**15 ± 2**	2.0 ± 0.2	1.9 ± 0.1
K11934	Membrane transport	outer membrane protein X	9090	23	73	**9 ± 2**	**11 ± 1**	**1.9 ± 0.2**	**1.4 ± 0.2**
K16553	Membrane transport	succinoglycan exporter	8372	15	84	**5 ± 2**	**9 ± 2**	1.3 ± 0.2	1.5 ± 0.4
K16348	Membrane transport	entericidin B	3282	22	77	**5 ± 1**	**10 ± 3**	**1.5 ± 0.2**	**1.9 ± 0.3**
K10975	Membrane transport	allantoin permease	1617	11	86	1 ± 1	1 ± 1	0.2 ± 0.4	0.1 ± 0.1
K16347	Membrane transport	entericidin A	1086	21	77	**3 ± 2**	**5 ± 1**	0.7 ± 0.5	1.1 ± 0.2
K11743	Membrane transport	spermidine export protein MdtJ	699	18	79	**1 ± 2**	**5 ± 0**	**0.3 ± 0.5**	**1.2 ± 0.2**
K16696	Membrane transport	exopolysaccharide (amylovoran) exporter	409	11	87	**0 ± 1**	**2 ± 1**	0.0 ± 0.1	0.3 ± 0.3
K12285	Biofilm formation	MSHA biogenesis protein MshO	25,345	27	70	**17 ± 3**	**24 ± 4**	2.5 ± 0.1	2.6 ± 0.1
K12280	Biofilm formation	MSHA biogenesis protein MshJ	20,898	26	70	**14 ± 1**	**20 ± 4**	**2.3 ± 0.1**	**2.1 ± 0.1**
K12284	Biofilm formation	MSHA biogenesis protein MshN	17,259	24	71	**10 ± 2**	**14 ± 2**	**1.8 ± 0.1**	**1.6 ± 0.1**
K10927	Biofilm formation	MSHA pilin protein MshD	15,335	27	69	**13 ± 2**	**21 ± 2**	**2.2 ± 0.2**	**2.6 ± 0.1**
K20961	Biofilm formation	diguanylate cyclase [EC:2.7.7.65]	14,724	21	77	**9 ± 1**	**11 ± 2**	**1.4 ± 0.3**	**1.1 ± 0.2**
K10926	Biofilm formation	MSHA pilin protein MshC	11,966	25	71	**13 ± 2**	**18 ± 2**	2.3 ± 0.2	2.4 ± 0.1
K12286	Biofilm formation	MSHA biogenesis protein MshP	11,500	26	71	**10 ± 2**	**16 ± 2**	**2.0 ± 0.2**	**2.4 ± 0.1**
K12281	Biofilm formation	MSHA biogenesis protein MshK	4887	23	74	**6 ± 1**	**9 ± 2**	1.5 ± 0.1	1.3 ± 0.2
K19731	Quorum sensing	LuxR family transcriptional regulator, quorum-sensing system regulator CciR	53,445	27	70	**37 ± 4**	**51 ± 7**	3.0 ± 0.2	3.1 ± 0.1
K20326	Quorum sensing	protein XagA	29,432	23	74	**17 ± 4**	**23 ± 3**	2.0 ± 0.2	2.2 ± 0.2
K20268	Quorum sensing	rhizosphere induced protein	10,459	25	72	**12 ± 4**	**23 ± 4**	**2.0 ± 0.4**	**2.4 ± 0.3**
K20267	Quorum sensing	type IV secretion system protein TrbH	3586	21	78	**4 ± 1**	**6 ± 2**	**1.1 ± 0.3**	**1.5 ± 0.3**
K20275	Quorum sensing	nematocidal protein AidA	2480	22	76	**4 ± 1**	**8 ± 2**	**1.1 ± 0.2**	**1.4 ± 0.2**
K20272	Quorum sensing	TraR antiactivator	1414	22	78	**3 ± 1**	**5 ± 1**	**0.8 ± 0.4**	**1.4 ± 0.3**
K03477	Transcriptional regulation	DeoR family transcriptional regulator, ulaG and ulaABCDEF operon transcriptional repressor	93,668	28	68	**49 ± 5**	**55 ± 5**	**3.1 ± 0.1**	**2.9 ± 0.1**
K21825	Transcriptional regulation	AraC family transcriptional regulator, L-arginine-responsive activator	31,901	25	71	**26 ± 3**	**32 ± 2**	2.7 ± 0.1	2.6 ± 0.2
K05372	Transcriptional regulation	AraC family transcriptional regulator	5092	24	74	**8 ± 3**	**14 ± 2**	**1.7 ± 0.4**	**2.1 ± 0.2**
K19060	Transcriptional regulation	TetR/AcrR family transcriptional regulator, macrolide resistance operon repressor	3198	22	76	**6 ± 2**	**11 ± 2**	1.5 ± 0.4	1.7 ± 0.2
K12820	Transcriptional regulation	pre-mRNA-splicing factor ATP-dependent RNA helicase DHX15/PRP43 [EC:3.6.4.13]	1417	11	87	**1 ± 1**	**2 ± 1**	0.1 ± 0.3	0.2 ± 0.2
K12059	Secretion systems	conjugal transfer pilus assembly protein TrbC	17,109	26	72	**18 ± 3**	**23 ± 6**	2.4 ± 0.2	2.5 ± 0.3
K12688	Secretion systems	autotransporter serine protease [EC:3.4.21.-]	10,969	18	80	**7 ± 2**	**16 ± 3**	**1.5 ± 0.2**	**1.9 ± 0.4**
K11017	Secretion systems	hemolysin activation/secretion protein	9861	24	73	**12 ± 3**	**17 ± 2**	2.2 ± 0.1	2.0 ± 0.3
K18380	Secretion systems	type III secretion control protein HpaB	5890	21	77	**3 ± 1**	**7 ± 3**	**1.0 ± 0.2**	**1.3 ± 0.3**
K12069	Secretion systems	conjugal transfer pilus assembly protein TraA	5753	23	75	9 ± 3	11 ± 1	1.9 ± 0.2	1.7 ± 0.2
K04338	Secretion systems	curli production assembly/transport component CsgF	4827	27	68	**9 ± 2**	**16 ± 3**	**2.0 ± 0.2**	**2.3 ± 0.3**
K18373	Secretion systems	type III secretion protein HrpB1	4535	23	75	**5 ± 1**	**8 ± 2**	1.3 ± 0.2	1.5 ± 0.3
K11889	Secretion systems	type VI secretion system protein ImpN [EC:2.7.11.1]	4012	15	85	**3 ± 2**	**7 ± 1**	**0.6 ± 0.5**	**1.3 ± 0.1**
K11909	Secretion systems	type VI secretion system protein VasI	2984	20	79	**6 ± 3**	**10 ± 3**	1.4 ± 0.4	1.6 ± 0.4
K03202	Secretion systems	type IV secretion system protein VirB7	2396	22	77	**5 ± 2**	**8 ± 3**	1.2 ± 0.5	1.6 ± 0.6
K12083	Secretion systems	type IV secretion system protein PtlH [EC:7.4.2.8]	1536	20	76	**3 ± 1**	**4 ± 0**	0.8 ± 0.3	0.6 ± 0.2
K12228	Secretion systems	TrbB protein	614	17	82	**1 ± 1**	**4 ± 2**	**0.1 ± 0.2**	**1.2 ± 0.4**
K20555	Secretion systems	type IV secretion system protein TrbK	613	21	79	**1 ± 1**	**3 ± 1**	**0.2 ± 0.3**	**0.9 ± 0.4**
K13454	Secretion systems	type III effector protein AvrRpm1	77	0	100	**0 ± 0**	**0 ± 1**	0.0 ± 0.0	0.0 ± 0.0
K19216	Antibiotic resistance	metallo-beta-lactamase class B IND [EC:3.5.2.6]	8968	18	80	**4 ± 2**	**9 ± 2**	**0.7 ± 0.4**	**1.2 ± 0.4**
K18793	Antibiotic resistance	beta-lactamase class D OXA-23 [EC:3.5.2.6]	6092	9	90	3 ± 1	3 ± 1	**0.5 ± 0.4**	**0.1 ± 0.0**
K19101	Antibiotic resistance	beta-lactamase class C FOX [EC:3.5.2.6]	5827	20	73	**6 ± 2**	**9 ± 1**	**1.4 ± 0.3**	**1.7 ± 0.2**
K22335	Antibiotic resistance	beta-lactamase class D OXA-114 [EC:3.5.2.6]	4627	22	76	**7 ± 3**	**13 ± 3**	**1.6 ± 0.5**	**2.0 ± 0.3**
K19213	Antibiotic resistance	beta-lactamase class D OXA-12 [EC:3.5.2.6]	1995	17	80	**3 ± 1**	**8 ± 2**	**0.7 ± 0.5**	**1.6 ± 0.2**
K02399	Motility	flagellar biosynthesis protein FlgN	22,038	24	72	**19 ± 3**	**28 ± 5**	**2.4 ± 0.2**	**2.1 ± 0.2**
K07345	Motility	major type 1 subunit fimbrin (pilin)	9256	25	72	**13 ± 4**	**23 ± 3**	2.2 ± 0.3	2.5 ± 0.2
K04643	Secondary messaging	sensory rhodopsin	6337	9	89	**4 ± 2**	**8 ± 2**	1.1 ± 0.5	1.0 ± 0.5
K20966	Secondary messaging	c-di-GMP phosphodiesterase [EC:3.1.4.52]	4168	19	79	**5 ± 1**	**7 ± 2**	**1.3 ± 0.2**	**1.6 ± 0.2**
K11964	Defence	pellino [EC:2.3.2.27]	2541	16	84	**3 ± 2**	**6 ± 2**	**0.7 ± 0.6**	**1.5 ± 0.4**
K13964	Defence	serpin B7	1767	16	79	3 ± 1	3 ± 1	**0.5 ± 0.2**	**0.2 ± 0.2**
K08566	Defence	plasminogen activator [EC:3.4.23.48]	1624	17	83	**2 ± 2**	**6 ± 1**	**0.5 ± 0.6**	**1.5 ± 0.2**
K01352	Defence	granzyme A [EC:3.4.21.78]	659	25	72	**2 ± 1**	**5 ± 2**	**0.5 ± 0.5**	**1.3 ± 0.4**
K13448	Calcium sensors	calcium-binding protein CML	1065	16	80	**1 ± 1**	**3 ± 1**	0.2 ± 0.2	0.3 ± 0.2
K10857	Cell cycle	exodeoxyribonuclease X [EC:3.1.11.-]	48,331	27	70	**26 ± 3**	**35 ± 5**	2.1 ± 0.2	2.2 ± 0.2
K13586	Cell cycle	holdfast attachment protein HfaB	29,948	26	72	13 ± 1	14 ± 2	**2.2 ± 0.1**	**2.0 ± 0.1**
K18642	Cell cycle	crescentin	10,803	22	78	**6 ± 1**	**7 ± 1**	1.3 ± 0.3	1.2 ± 0.2
K13585	Cell cycle	holdfast attachment protein HfaA	8477	19	81	**7 ± 1**	**10 ± 1**	**1.7 ± 0.2**	**2.0 ± 0.2**
K14781	Cell cycle	ATP-dependent RNA helicase DHR2 [EC:3.6.4.13]	1029	8	91	**0 ± 0**	**1 ± 0**	0.0 ± 0.0	0.0 ± 0.0
K13831	Primary metabolism	3-hexulose-6-phosphate synthase/6-phospho-3-hexuloisomerase [EC:4.1.2.43 5.3.1.27]	2008	56	18	**3 ± 1**	**1 ± 1**	**1.0 ± 0.2**	**0.1 ± 0.3**
K00608	Primary metabolism	aspartate carbamoyltransferase [EC:2.1.3.2]	319	88	10	**2 ± 1**	**0 ± 0**	**0.7 ± 0.5**	**0.0 ± 0.0**
K21346	Primary metabolism	methionine transaminase [EC:2.6.1.88]	311	62	14	**2 ± 1**	**0 ± 0**	**0.5 ± 0.3**	**0.0 ± 0.0**
K05660	Membrane transport	ATP-binding cassette, subfamily B (MDR/TAP), member 5	136	74	7	1 ± 1	0 ± 0	0.1 ± 0.2	0.0 ± 0.0
K07991	Motility	archaeal preflagellin peptidase FlaK [EC:3.4.23.52]	602	22	4	**1 ± 1**	**0 ± 0**	0.2 ± 0.2	0.0 ± 0.0
K02383	Motility	flagellar protein FlbB	55	97	1	**0 ± 1**	**0 ± 0**	**0.1 ± 0.2**	**0.0 ± 0.0**

Bold indicates Welch Two Sample tests (*p* < 0.05) using a false-discovery rate correction to adjust for multiple comparisons

The reads assigned to the differentially abundant functional genes between heritage and modern rhizospheres were re-processed though our bioinformatic pipeline and assigned to taxa. This allowed us to look for taxonomic differences and potential important taxa for these functions. Out of the 107 functional genes enriched in heritage rhizospheres, 87% had higher taxa richness in the heritage rhizospheres, but only 37% had higher taxa diversity (Shannon’s diversity). This suggests that evenness in these communities may be important whereby dominant taxa could be driving these differences in function (Table 3). This was further evident by the differences in the community composition, based on the relative abundance of phyla, between heritage and modern rhizospheres for each differentially abundant gene (Table S2). In the heritage rhizospheres the dominant phyla or class often assigned to a higher proportion of reads than in the modern rhizospheres, except where a function was only carried out by taxa from a single phylum.

## Discussion

We compared the shotgun metagenomic profiles of prokaryote communities from the rhizospheres of pre- (heritage) and post-green revolution (modern) wheat cultivars and found clear differences between these groupings. We also confirmed our hypothesis that taxonomic changes relate to changes in the potential function of these communities, with the main difference being a comparative depletion of many functional genes in modern wheat rhizospheres. Furthermore, the addition of unplanted bulk soil controls in our study confirms that the rhizosphere microbiome of modern cultivars differentiate less from bulk soil than those derived from heritage cultivars in terms of both taxonomy and function. These observations evidence that wheat breeding during the green revolution have resulted in cultivars with reduced ability to select a rhizosphere microbiome from the bulk soil microbial reservoir.

### Taxonomic differences

When using shotgun metagenomics instead of phylogenetic marker gene analysis, we identified tenfold more differentially abundant prokaryote taxa between heritage and modern cultivars than in our previous study [19]. Both studies found that most differentially abundant taxa belonged to Pseudomonadota, Bacteroidota and Actinomycetota and were enriched in heritage cultivars, though our results showed 95% enrichment compared with 69% previously. We also found higher species richness in modern rhizospheres, supporting their reduced selectivity, while Shannon's diversity was higher in heritage rhizospheres in our study but not in Kavamura et al. [19]. These minor discrepancies could result from additional cultivars or different edaphic factors and growing conditions between studies.

Reid et al. [51] found that chemical fertilization impacts root microbiome structure regardless of plant genotype. However, we used fertilized soil in our study to reflect common wheat growing conditions and because polyploid wheats showed reduced capacity to select for plant growth promoting bacteria despite fertilization treatment. Our results also supported this study by demonstrating a loss of Bacteroidota in modern cultivars in both relative abundance and differentially abundant taxa. This is also consistent with other wheat domestication studies [1, 47, 53]. Bacteroidota are important for plant pathogen protection and phosphorus uptake [26]. Domestication has also been shown to enrich Actinobacteria and phyla commonly associated with bulk soil, such as Acidobacteriota and Verrucomicrobiota [1, 47, 53, 19]. By including unplanted bulk soil controls, our study confirms that modern wheat rhizosphere microbiomes more closely resemble bulk soil than heritage cultivars, indicating less filtering of prokaryote taxa in modern varieties.

### Functional gene differences

#### Bulk soil vs rhizosphere soils

As described in the results section, 19 of the 20 most abundant genes that are enriched in heritage rhizospheres compared to bulk soils are also more abundant in heritage than modern rhizospheres, but to a lesser extent (Table S1). This suggests that the selective ability of modern cultivars is diminished in comparison to heritage cultivars, and the rhizosphere microbiomes in modern are a ‘middle ground’ between bulk soil and heritage rhizospheres (Fig. 1d). The exception was K07305 (peptide-methionine (R)-S-oxide reductase), which showed equal abundance in heritage and modern rhizosphere microbiomes but was significantly higher than in bulk soil (approximately 500 K mean normalized reads per rhizosphere sample versus 48 K per bulk soil sample). This enzyme repairs oxidatively damaged proteins. We hypothesize that its high abundance in the rhizosphere reflects intense metabolic activity and consequent free radical generation. The prevalence of this gene appears crucial for rhizosphere competence regardless of wheat genotype. Future studies could determine whether expression of this gene is essential for microbial colonization of the plant-root environment.

#### Heritage vs modern rhizospheres

Shotgun metagenomic analysis of samples from the rhizosphere of heritage and modern wheat cultivars resulted in the detection of clear differential abundance in reads mapping to 113 genes. With 107 genes enriched in the rhizospheres of heritage wheats, these results possibly indicate that the genetic potential of the host plant to influence the root microbiome structure and function has been reduced as a consequence of wheat breeding during the green revolution. We discuss the functions associated with genes enriched in the rhizospheres of heritage wheats (see KEGG IDs in brackets) under 11 major categories below in order of highest read counts allocated per category. Not all 107 genes are discussed in detail but see Table 3 and Table S2 for a summary in terms of read counts and taxonomic information. The six genes enriched in the modern rhizosphere samples assign to primary metabolism, membrane transport and motility, but in much fewer read counts (3 K reads) than those enriched in heritage cultivars (6.5 M reads).

##### Secondary metabolism

There were 13 differentially abundant genes relating to secondary metabolism with a total of 2.6 M reads assigned over all samples. Of these reads, 98% were most similar to genes associated with biosynthesis of staurosporine (K14266, K19885, K20086, K20090 and K20088), a natural product antibiotic originally isolated from the bacterium *Streptomyces staurosporeus* [43], with mode of action being through competitive protein kinase inhibition, with this family of molecules exhibiting anti-cancer potential [70]. In addition, reads were associated with terpenoid and polyketide antibiotic synthesis (K16397, K16403, K14627, K14368, K21212, K16448 and K15968) as well as bacterial toxin production (K11009). The high number of differentially abundant genes associated with antibiotic production could indicate that niche occupancy competition of rhizosphere microbiome community members in pre-green revolution wheat is driven by an arms race, which could provide a novel underexploited resource for natural product discovery and the development of the next generation of antibiotics.

##### Cell wall degradation

A total of 1.3 M reads were found to map to four differentially abundant genes for plant cell wall degradation (K18651, K18578, K18576 and K18786). Although this function has long been associated with plant pathogen function [22, 69], cellulolytic activity has also been linked to enhanced plant root length by facilitating the sloughing-off of root cap cells from root tips which assists the growing root in penetrating soil [10]. It has also been found that cell wall degradation is essential for *Rhizobium* symbiotic infection of legume roots [52]. It follows that this function could also be important for microbial colonisation of the plant environment, microbial mediation of plant root architecture and access to an overwintering energy source for microbes on crop residues. With such high enrichment of these genes in heritage wheat rhizospheres therefore demonstrates that a potentially important function to facilitate plant-microbial interactions has been degraded in modern wheat.

##### Primary metabolism

In addition to cell wall degradation, a further 809 K reads mapped to genes associated with primary metabolism. These can primarily be subdivided into the metabolism of carbohydrates (380 K reads), lipids (340 K reads), proteins (46 K reads), co-factors/vitamins (27 K reads), and enzymes involved in core primary metabolism (15 K reads). These observations are congruent with the classes of genes most abundant in membrane transport. The most abundant of the differentially abundant primary metabolism genes was associated with sphingolipid metabolism through the action of galactosylceramidase (K01202, 336 K reads). Sphingolipids are a class of membrane bound lipid which act as signal bioactive molecules [67]. It has been proposed that Bacteroidota, predominant members of the mammalian gut microbiome, utilise sphingolipids as an energy source as well as to mediate signal transduction and stress response pathways, facilitating their persistence in this environment [2]. Our dataset found that Actinomycetota made up the highest proportion of taxa with this gene (47%) demonstrating it is not a Bacteroidota specific function in the wheat root environment. It could be the case that microbe-microbe and microbe-plant sphingolipid-based signalling is also crucial in commensal colonisation of the plant root environment as is proposed in the human gut.

##### Sensor regulator

Approximately 640 K reads were found to map to differentially abundant genes associated with two component sensor-regulation systems, most of which (510 K reads) were assigned to serine detection (K05874). In addition, a sensor receptor gene for monosaccharides (ribose and galactose; K05876) was also in high abundance. It will be interesting in future work to determine the amino acid to sugar ratio and the relative contribution of amino acids in the root exudates of heritage compared to modern wheats and determine whether detection of these molecules is important for chemoattraction into the root zone as a prerequisite to rhizosphere colonisation of heritage wheat.

##### Membrane transport

A total of 520 K reads mapped to differentially abundant genes associated with membrane transport and the vast majority (435 K reads) were associated with uptake systems. However, export systems were largely associated with polysaccharide transport, presumably as a prerequisite to biofilm formation (e.g. K16552, K16553, K16696). Regarding import systems, these were categorised as metal (iron and nickel; K10094, K16088), purine (K10975), aromatic amino acids (K11734), though the vast majority of these reads were associated with ATP-binding cassette and MFS sugar uptake systems (alpha-glycosides, arabinose, lactose and oligogalacturonides) with a total of 280 K reads assigned (K02532, K10235, K16210, K08156). The observation that bacteria in the rhizosphere have a high level of uptake transport systems has previously been studied in rhizobia [34], though this is the first time that an enhancement in these systems is associated with the root microbiome of pre-green revolution wheats. It is interesting that there was no perceived difference in abundance detected between heritage and modern wheats for genes associated with organic acid uptake systems and implies that the green revolution development of wheat has perhaps not impacted the root exudation profile of these molecules to the same extent for amino acids and sugars. Furthermore, a recent study by [32], suggested that plants also exude hemicelluloses. Although there is not an apparent enrichment in glycan importing TonB-dependent transporter genes in our data, the high abundance of genes encoding xyloglucan degrading enzymes implies that breakdown products of these molecules could be imported via high affinity systems.

##### Biofilm formation and quorum sensing

Approximately 234 K reads mapped to genes associated with quorum sensing and biofilm formation. Reads mapping to genes associated with mannose sensitive haemagglutinin (MSHA) pilus biogenesis [*mshJ*, (K12280) *mshK* (K12281), *mshN* (K12284), *mshO* (K12285) and *mshP* (K12286)] are total 83 K reads. This molecule has been shown to be crucial for the attachment of microbes to surfaces (Marsh and Taylor, 1999; Dalisay et al., 2006). It therefore follows that the increased differential abundance of MSHA could reflect their importance for microbial persistence in the heritage wheat rhizosphere microbiome.

In addition, approximately 106 K reads mapped to genes associated with quorum sensing. The most abundant of these, at 56 K reads, being assigned as the LuxR family transcriptional regulator gene *cciR* (K19731) which has previously been implicated in exopolysaccharide (EPS) synthesis and controlling biofilm formation [74]. Our data suggests that transcriptional regulators are also important for biofilm formation in the root environment and emphasised in pre-green revolution wheat.

##### Transcriptional regulation

Approximately 142 K reads were assigned to transcriptional regulation, and almost 100 K are ascribed to the DeoR family transcriptional regulator (K03477). In *E. coli*, a DeoR family transcriptional regulator, UlaR, was found to be responsible for suppressing transcription of the divergent *ulaG* and *ulaABCDEF* operons (which catabolise L-ascorbate), under ascorbate depleted conditions [11]. Interestingly, ascorbate has been shown to be released from plant roots under conditions of salt stress and influences root elongation [25, 31]. It could follow that exudation of ascorbate into the rhizosphere profoundly affects root microbiome colonisation patterns in heritage wheat cultivars.

##### Secretion systems

A total of 76 K reads mapped to secretion systems, and interestingly, type III (K13454, K18380, K18373, K18380), IV (K03202, K12083, K120555) and type VI (K11889, K11909) secretion systems are predominately overrepresented in heritage wheat rhizospheres, all of which use machinery to directly breach and deliver secreted proteins across host cell membranes as opposed to other secretion system types which release toxins into the extracellular milieu [16].

##### Antibiotic resistance

Approximately 29 K reads were assigned to differentially abundant genes involved in antibiotic resistance (K19216, K18793, K19101, K22335 & K19213). These were related to beta-lactamase function, which has been shown to be well represented in isolates of the soil dwelling bacterium *Bacillus subtilis* [5]. Evidence suggests that its production facilitates rhizosphere colonisation by the plant pathogen *Fusarium oxysporum* [12]. It is interesting that the number of reads in this category is far exceeded by the number of reads for antibiotic production (e.g. staurosporine biosynthesis). This could indicate that microbial strategy for rhizosphere colonisation of heritage wheat rhizospheres have an offensive emphasis.

##### Motility

There were two differentially abundant genes relating to motility, but the majority of the reads (23 K reads) were assigned to the flagellar chaperone biosynthesis gene *flgN* (K02399). This gene has been shown to be required for flagellum-based motility in *Bacillus subtilis* [9] and is involved in the regulation and assembly of the flagellum, and its enhanced differential abundance in the rhizosphere of heritage wheat suggests that it is important for the colonisation of this environment.

##### Secondary messaging

Approximately 10 K reads were assigned to secondary messaging, especially cyclic di GMP (c-di-GMP) phosphodiesterase (K20966) which has previously been shown to be important for the rhizosphere colonisation of wheat by *Pseudomonas fluorescens* [27]. The activity of c-di-GMP is important for the production of EPS and biofilm formation.

## Conclusions

Our data showed that modern wheat has a reduced rhizosphere effect when compared with heritage wheat. In addition, we observed a depletion of a wide range of functional genes in modern wheat, indicating a functional deterioration in the rhizosphere microbiome associated with the green revolution. Wheat breeding during the green revolution has profoundly influenced the selection and function of the root microbiome, and this is evidenced by reduced abundance in genes such as those involved in secondary metabolism as well as cell wall degradation. As such it seems that the root microbiome of heritage wheats have adopted a two-pronged strategy of exclusion of microbial competitor reduction through antibiotic production (e.g. staurosporine production) as well as the metabolism of plant derived nutrients. The latter seems to be via utilisation of cell wall constituents as a nutrient source to establish in this environment as well as through sphingolipid metabolism – it is unclear to what extent the latter function is also important for plant–microbe signalling. The green revolution combined wheat dwarfing with the application of synthetic chemical fertilisers. Our previous work has highlighted that application of synthetic fertiliser reduces the selection of nutrient-solubilising bacteria in the rhizosphere [50]. It will be interesting to ascertain the impact of the combination of these factors for global microbiome function, and to determine the specific contribution of *Rht* mutant alleles, responsible for wheat dwarfing, by studying the microbiome of isogenic wheats. Finally, we believe our results are of striking importance and highlight that if implementation of microbiome facilitated agriculture is to be introduced as part of a sustainable crop production strategy an overhaul of wheat breeding programmes will be necessary to consider plant–microbe interactions, especially in the root environment.

## Supplementary Information

Additional file1

## Data Availability

The genomic datasets generated and analysed during the current study are available in the NCBI repository, BioProject PRJNA1128034. The BioProject and associated SRA metadata are publicly available at https://www.ncbi.nlm.nih.gov/bioproject/PRJNA1128034. R scripts and associated data for all tables and figures in this manuscript are available on GitHub at https://github.com/MoniqueSmithSLU/Wheat_metagenomes.
